# Editorial: Opportunities and challenges in reusing public genomics data

**DOI:** 10.3389/fphar.2023.1226756

**Published:** 2023-06-12

**Authors:** Mahmoud Ahmed, Deok Ryong Kim

**Affiliations:** Department of Biochemistry and Convergence Medical Sciences, Institute of Health Sciences, College of Medicine, Gyeongsang National University, Jinju, Republic of Korea

**Keywords:** reusing public data, genomics, data sharing, metadata, data curation and integration

Genomics data is accumulating in public repositories at an ever-increasing rate. Large consortia and individual labs continue to probe animal and plant tissue and cell cultures, generating vast amounts of data using established and novel technologies. The human genome project kick started the era of systems biology (Lander et al., 2001; Gates et al., 2021). Ambitious projects followed to characterize non-coding regions, variations across species, and between populations (Feingold et al., 2004; Sabeti et al., 2007; Auton et al., 2015). The cost reduction allowed individual labs to generate numerous smaller high-throughput datasets (Edgar et al., 2002; Parkinson et al., 2007; Metzker, 2010; Leinonen et al., 2011). As a result, the scientific community should consider strategies to overcome the challenges and maximize the opportunities to use these resources for research and the public good. In this Research Topic, we have elicited opinions and perspectives from researchers in the field on the opportunities and challenges of reusing public genomics data. The articles in this Research Topic converge on the need for data sharing while acknowledging the challenges that come with it. Two articles defined and highlighted the distinction between data and metadata. The characteristic of each should be considered when designing optimal sharing strategies. One article focuses on the specific issues surrounding the sharing of genomics interval data, and another on balancing the need for protecting pediatric rights and the sharing benefits.

The definition of what counts as data is itself a moving target. As technology advances, data can be produced in more ways and from novel sources. Events of recent years have highlighted this fact. “The pandemic has underscored the urgent need to recognize health data as a global public good with mechanisms to facilitate rapid data sharing and governance,” Schwalbe et al. The challenges facing these mechanisms could be technical, economic, legal, or political. Defining what data is and its type, therefore, is necessary to overcome these barriers because “the mechanisms to facilitate data sharing are often specific to data types.” Unlike genomics data, which has established platforms, sharing clinical data “remains in a nascent phase.” The article by Patrinos et al. considers the strong ethical imperative for protecting pediatric data while acknowledging the need to avoid over protections. The authors discuss a model of consent for pediatric research that can balance the need to protect participants and generate health benefits.

Xue et al. focus on reusing genomic interval data. Identifying and retrieving the relevant data can be difficult, given the state of the repositories and the size of these data. Similarly, integrating interval data in reference genomes can be hard. The author calls for standardized formats for the data and the metadata to facilitate reuse.

Sheffield et al. highlight the distinction between data and metadata. Metadata describes the characteristics of the sample, experiment, and analysis. The nature of this information differs from that of the primary data in size, source, and ways of use. Therefore, an optimal strategy should consider these specific attributes for sharing metadata. Challenges specifics to sharing metadata include the need for standardized terms and formats, making it portable and easier to find.

We go beyond the reuse issue to highlight two other aspects that might increase the utility of available public data in Ahmed et al. These are curation and integration. Despite being generated using different protocols, combining the datasets from separate groups could help to fill the gaps in the design and increase the statistical power of the analysis. Integrating data types can be beneficial to either verify or complement the observations made based on a single data type. We also emphasize the critical requirements for these strategies to be successful. We draw on our experience and others in using publicly available datasets to support, develop, and extend our research interest.

The articles in this Research Topic converge on the importance of data sharing. In addition, the articles present the challenges facing data sharing and reuse and propose models to increase the utility of public data.
